# Supplementary material for: Evaluation of Liver Quality after Circulatory Death versus Brain Death: A Comparative Preclinical Pig Model Study
Source: Int J Mol Sci. 2020 Nov 27;21(23):9040. doi: 10.3390/ijms21239040 (PMC7730280; doi:10.3390/ijms21239040)
Supplement: Supplementary file 1 [file ijms-21-09040-s001.pdf]

Supplementary Table 1 : RTqPCR Primer sequences

Target	Forward Primer	Reverse Primers
ATG7	GGGTCTTCTGACTTGGAAGC	CACAGCAGTGAGGACCAGAC
β-Actin	GATCGTGCGGGACATCAAG	GCCATCTCCTGCTCGAAGTC
BCL XL	GCTTTGAGCAGGTAGTGAACG	CCCACCGAAGGAGAAAAAG
CLUSTERIN	TGGCCCCTGGGTGCT	CCTCCTTGAGGGCATTTTT
CYA62	AAGACTGAGTGGTTGGATGG	AATGGTGATCTTCTTGCTGGT
EPO	TGCACAACTGTGATGCCTGGAC	TCCATTCTGTCCCTGCTTGGTAGG
GLUT1	GACCTTCGATGAGATTGCTTCCG	TCGGGTGTCTTGTCGCTTTG
HBEGF	GCAGACCTGGACCTTTTGAG	CTTTTCCCTCGCTCCTCCT
HGF	TCATTCCACTCCCAACCACT	TGTGTTTCTTCTTTTCTTCTGTCC
HIF1a	TGGCAGCAATGACACAGAAAC	GAGGCAGGCAATGGAGACAT
HO1	ACTCAGCTTTCTGGTGGCAACG	CACAGGGTTGCATTCACATGGC
L19	AATCGCCAACGCCAACTC	CAGCCCATCTTTGATCAGCTT
MCL1	AGGACAAAACGAGACTGGCTA	ATTTCTGATGCCGCCTTCTA
NOTCH4	CAGTCCAAGCAAAAATGCCAG	GTGTGGAGATGCAGCCGC
NOX2	GTGCACCATGATGAGGAGAA	AGTTAGGCCGTCCGTACAAG
RPLPO	AGAAACTGTTGCCTCACATCC	CCTTATTGGCCAGCAGCA
SDHA	GAGTTCGTGCAGTTCCACCCTA	CCTCTCACCCTGGCTGTTGATA
SOD2	TGAACAACCTGAACGTCGTG	AGGCTGCAGAGCTACCTGAG
TGFα	TGGTGGTGGTCTCCATAGTG	GGCACCACTCACAGTGTTTT
TGFβ	CGAGCCAGAGGCGGACTA	TATCATAGATTTGGTTGCCGCTTT
TNFα	TCAGATCATCGTCTCAAACCTC	CCAGCTGGTTGTCTTTCAGC
WNT4	CCCACCTGGCGGAAG	CATGCGGGTGGAGTGC

Supplementary Methods

Experimental model:

Animal experiments were conducted at the IBiSA platform 'Experimental Surgery and Transplantion' in Surgères, France, in accordance with the regulations of French Ministry of Agriculture and National Institute for Agronomic Research and ethical regional committee on animal experiments (Protocol #CE2012-11). This facility provides near-clinical conditions for both surgery and ICU. We used 3 months old Large White male pigs (45-55kg). Anesthesia was performed as previously published [1].

Anesthesia [1]**:**

Anesthetic induction of the animal was performed with a Hunter mask with a 50/50 mixture of nitrogen protoxyde and oxygen associated with 8% sevoflurane; deepened with an injection of propofol 2.5 mg/kg, midazolam 0.5 mg/kg, fentanyl 10 mcg/kg and rocuronium bromure 1 mg/kg. The animal was scoped and perfused at a marginal vein of the ear. Oro-tracheal intubation was performed under laryngoscopic control. Controlled ventilation was managed with a tidal volume of 10 ml/kg, a respiratory rate of 16/min and maintaining an End-tidal CO_2 expired between 35 and 45mmHg and positive expiratory pressure of 5 cmH_2O. Curarization of the animal was performed by reinjections adapted to the behavior of the animal with rocuronium bromure 1 mg/kg. Intraoperative analgesia was maintained by continuous infusion of fentanyl 10 mcg/kg/h. Anesthesia was maintained by isoflurane 2.5%.

ANOR model [2,3]:

A Rotaflow Maquet centrifugal pump with Softline Coating Maquet-Jostra pre-heparined circuit (Maquet Cardiopulmonary AG) was used with athermoregulator system to maintain 37°C. The circuit was primed with 700 to 800cc hydroxylethyamidon (Restorvol® 6%; Fresenius Kabi). For surgery, animals was placed supine with access to cervical, thoracic and abdominal regions. First, a central venous catheter was positioned in the internal jugular vein after right cervicotomy. Then, laparotomy was performed. The abdominal aorta and inferior vena cava under the renal vessels were dissected since femoral vessels are too small to accomodate cannulae of sufficient size to ensure the target flow rate. We choose an extra-peritoneal approach to avoid lymphatic losses to limit vascular filling and hemodilution, as the formation of a third sector is frequently encountered in renal transplants performed by a trans-peritoneal approach in the laboratory. A bolus of 300UI/kg of unfractionated heparin (UFH) (Sanofi-Aventis, Paris, France) was injected through the central venous catheter as recommended by the French Biomedicine Agency, to limit the activation of coagulation on the plastic of the extracorporeal pump. After 5 minutes, a 14Fr arterial cannula (Medtronic Inc) was placed in the abdominal aorta under the renal arteries and a 21Fr long multiperforated venous cannula (Medtronic) was positioned at the junction of the inferior vena cava and the right auricle. Cannulae were introduced according to the Seldinger technique to be completely sealed. Circulatory arrest was induced by the injection of 2g potassium chloride through the central venous catheter then the mechanical ventilation was stopped.

The descending thoracic aorta was approached by a short left thoracotomy so as to be clamped. Another catheter was placed downstream of the clamp to monitor blood pressure. After 30 minutes of WI, ANOR was started for 4h (DCD group) before organ collection

Brain death model [4]:

Cortical activity was measured by 5 subcutaneous electrodes permitting a continuous two-derivation electroencephalogram. Hemodynamic reanimation consisted in an iterative volume compensation based on volemic measurements and the administration, as needed, of noradrenalin associated with 50mg hydrocortisone hemisuccinate to maintain a mean arterial pressure of 65 mmHg. In case of alteration of the cardiac rhythm during brain death, animals were injected with 300mg amiodarone.

An incision was performed to uncover the perioste. A 2cm in diameter trepanation was executed using a hand-driven neurosurgical drill fitted first with a triangular drill bit then a soft tipped drill bit in order to uncover the extra dural space. The dura matter was carefully detached using an atraumatic rugine, a urinary probe was inserted in the extradural space and the orifice was closed with orthopedic acrylic cement to insure impermeability. Brain death was reached by progressive insufflation of the balloon using a manual insufflator (Encore26, Boston Scientific) until a flat electroencephalogram was obtained. Reanimation was maintained for 4 hours before organ collection.

Organ collection:

At the end of the procedure, or immediately after anesthesia for the control group, the animal were laparotomized and liver vessels were rapidly cannulated (aorta and vena cava). After systemic injection of heparin (UI), the thoracic aorta was clamped, the abdomen iced with 4°C NaCl and sterile ice, and the liver was flushed with 5L of Histidine Tryptophane Ketoglutarate solution. At the end of the flush, the liver was collected and preserved in 500mL of HTK in an ice chest.

Experimental design and sample collection (Supplementary Figure 1):

Livers collected after each of the three protocols and kept at 4°C during a period of 24 hours. Organs were biopsied at regular intervals to monitor the state at the time of preservation and evolution of this state with preservation time. Experimental times were 0, 2, 6, 8 and 24 hours of preservation, with either formalin fixed samples (anatomopathology) or snap frozen samples (RTqPCR, Western blot, TUNEL and CellRox assays).

Anatomopathological analysis:

Formalin-fixed samples were embedded in paraffin and processed for anatomopathologic evaluation. HES and PAS stained slides were evaluated by a blinded pathologist, semi-quantitatively evaluating lesions markers was performed as detailed in Supplementary Figure 2.

Real-Time Quantitative PCR:

Total RNAs from liver tissue were extracted with a commercial kit (Macherey-Nagel, Hoerdt, France). Genomic DNA was removed using DNA-free kit (Applied-Biosystems, Saint-Aubin, France) and first-strand reverse transcription (Applied-Biosystems, Saint-Aubin, France) was performed. Real-Time qPCR assays were performed on a RotorGene Q (Qiagen, Courtaboeuf, France) with SYBR green

technology (Life Technologies) following the manufacturer's recommendations. Porcine DNA primers were designed using OligoPerfect™ (Life Technologies, Saint Aubin, France), QuantPrim (Universität Potsdam, Max-Planck-Gesellschaft, Potsdam, Germany) and OligoAnalyser (Integrated DNA Technologies, Coralville, Iowa, USA) with the sequences detailed in Supplementary Table 1. All primers were validated, demonstrated efficiency between 1.8 and 2.2. mRNA expression levels in the samples relative to expression in healthy kidney were determined with the Pfaffl method [5] (expressed as Relative Fold Change), using ribosomal L19, β-Actin, SDHA, CYA62 and RPLPO genes as internal controls. Healthy kidney were chosen as normalization in order to gauge alterations in the Control group.

Western blot analysis:

Western Blotting was carried out according to standard protocols, loading 30µg of protein per lane, using specific antibodies against: Hypoxia Inducible factor 1 alpha (HIF1α, BD Bioscience, France), Superoxyde Dismutase (SOD) 1 (Santa Cruz, USA) and 2 (Cell Signaling, USA), Heme oxygenase 1 (HO-1, Bioss, France), Catalase Bcl2, Bax (all from Cell Signaling, USA), Erythropoietin (EPO, AbGent , France), 4-Hydroxynonenal (4 HNE) and nitrotyrosine (N-Tyr, both from Millipore, France). Migration, transfer and quantification were performed using the criterion, transblot and chemidoc technologies (Biorad, Marnes La Coquette, France), using the stain free technology to normalize the signal between the lanes (Image Lab software, biorad). Stain free was favored over classical loading controls such as βactin and GAPDH as these proteins have been demonstrated to be affected by ischemia reperfusion and thus do not fulfill the necessary requirements for a loading control (i.e.: stability over the experimental conditions).

TUNEL Staining:

TdT-mediated dUTP Nick-End Labeling assay was performed on frozen samples using the DeadEnd™ Fluorometric TUNEL System (Promega, Charbonnieres, France) according to the manufacturer's recommendations. Ratio of TUNEL positive cells over the total number of cells was evaluated on 5-10 high powered fields (200X) using ImageJ (NIH, Bethesday, USA)

CellRox Staining:

Production of reactive oxygen species was measured on frozen samples using CellROX® Green Reagent (Fisher Scientific, Illkirch, France) using the manufacturer's recommendation. Signal quantification was performed on 10-15 high powered fields (200X) using ImageJ.

1. Soussi, D.; Danion, J.; Baulier, E.; Favreau, F.; Sauvageon, Y.; Bossard, V.; Matillon, X.; Turpin, F.; Belgsir, E.M.; Thuillier, R.; et al. Vectisol Formulation Enhances Solubility of Resveratrol and Brings Its Benefits to Kidney Transplantation in a Preclinical Porcine Model. *Int. J. Mol. Sci.* **2019**, *20*, doi:10.3390/ijms20092268.
2. Allain, G.; Kerforne, T.; Thuret, R.; Delpech, P.-O.; Saint-Yves, T.; Pinsard, M.; Hauet, T.; Giraud, S.; Jayle, C.; Barrou, B. Development of a preclinical model of donation after circulatory

determination of death for translational application. *Transplant. Res.* **2014**, *3*, 13, doi:10.1186/2047-1440-3-13.
3. Kerforne, T.; Allain, G.; Giraud, S.; Bon, D.; Ameteau, V.; Couturier, P.; Hebrard, W.; Danion, J.; Goujon, J.-M.; Thuillier, R.; et al. Defining the optimal duration for normothermic regional perfusion in the kidney donor: A porcine preclinical study. *Am. J. Transplant. Off. J. Am. Soc. Transplant. Am. Soc. Transpl. Surg.* **2018**, doi:10.1111/ajt.15063.
4. Kerforne, T.; Giraud, S.; Danion, J.; Thuillier, R.; Couturier, P.; Hebrard, W.; Mimoz, O.; Hauet, T. Rapid or Slow Time to Brain Death? Impact on Kidney Graft Injuries in an Allotransplantation Porcine Model. *Int. J. Mol. Sci.* **2019**, *20*, doi:10.3390/ijms20153671.
5. Bustin, S.A.; Wittwer, C.T. MIQE: A Step Toward More Robust and Reproducible Quantitative PCR. *Clin. Chem.* **2017**, *63*, 1537–1538, doi:10.1373/clinchem.2016.268953.

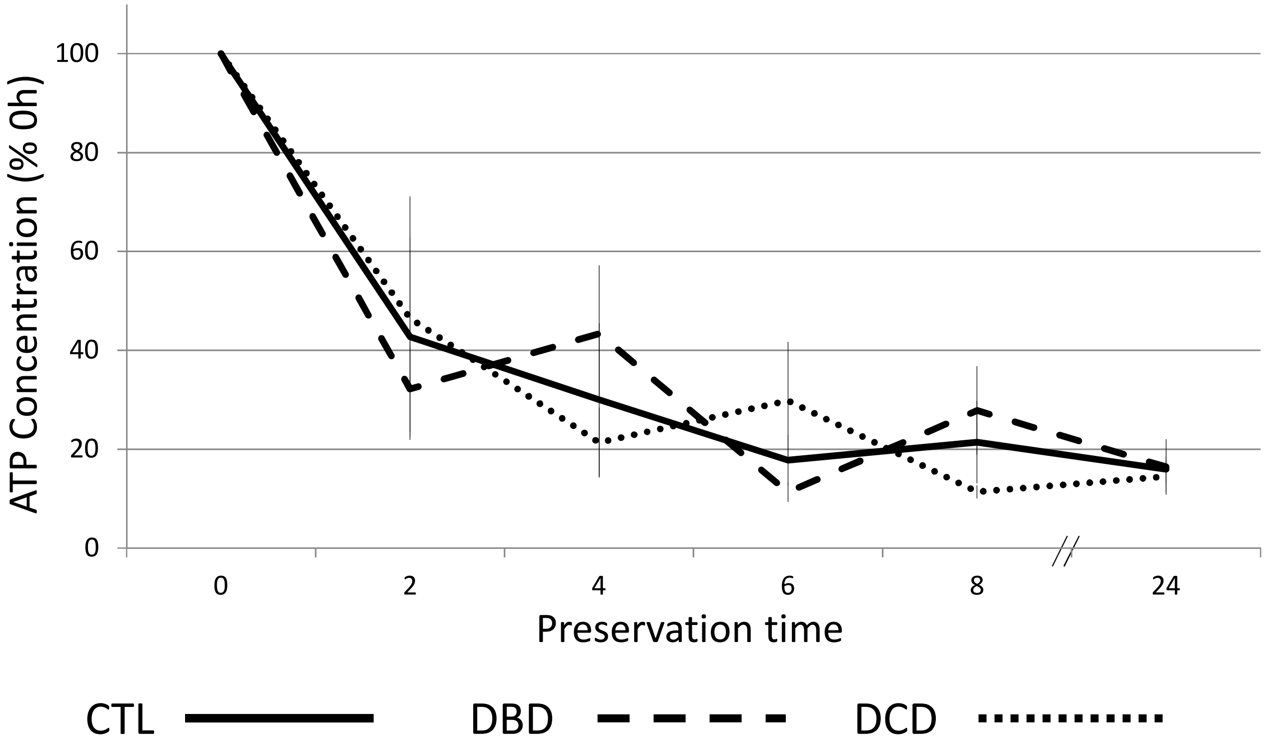

Supplementary Figure 1: ATP quantification during preservation
Liver biopsies were immediately frozen in liquid nitrogen at the indicated times and processes for ATP quantification using the Adenosine 5′-triphosphate (ATP) Bioluminescent Assay Kit from Sigma Aldrich (St. Quentin Fallavier, France). ATP concentration were normalized to the nucleotide content of the sample, measured by NanoDrop spectrometry. Results are shown as percentage of ATP detected at 0h, to express evolution of ATP during preservation. CTL group: solid line; DBD group: dashed line; DCD group: dotted line.

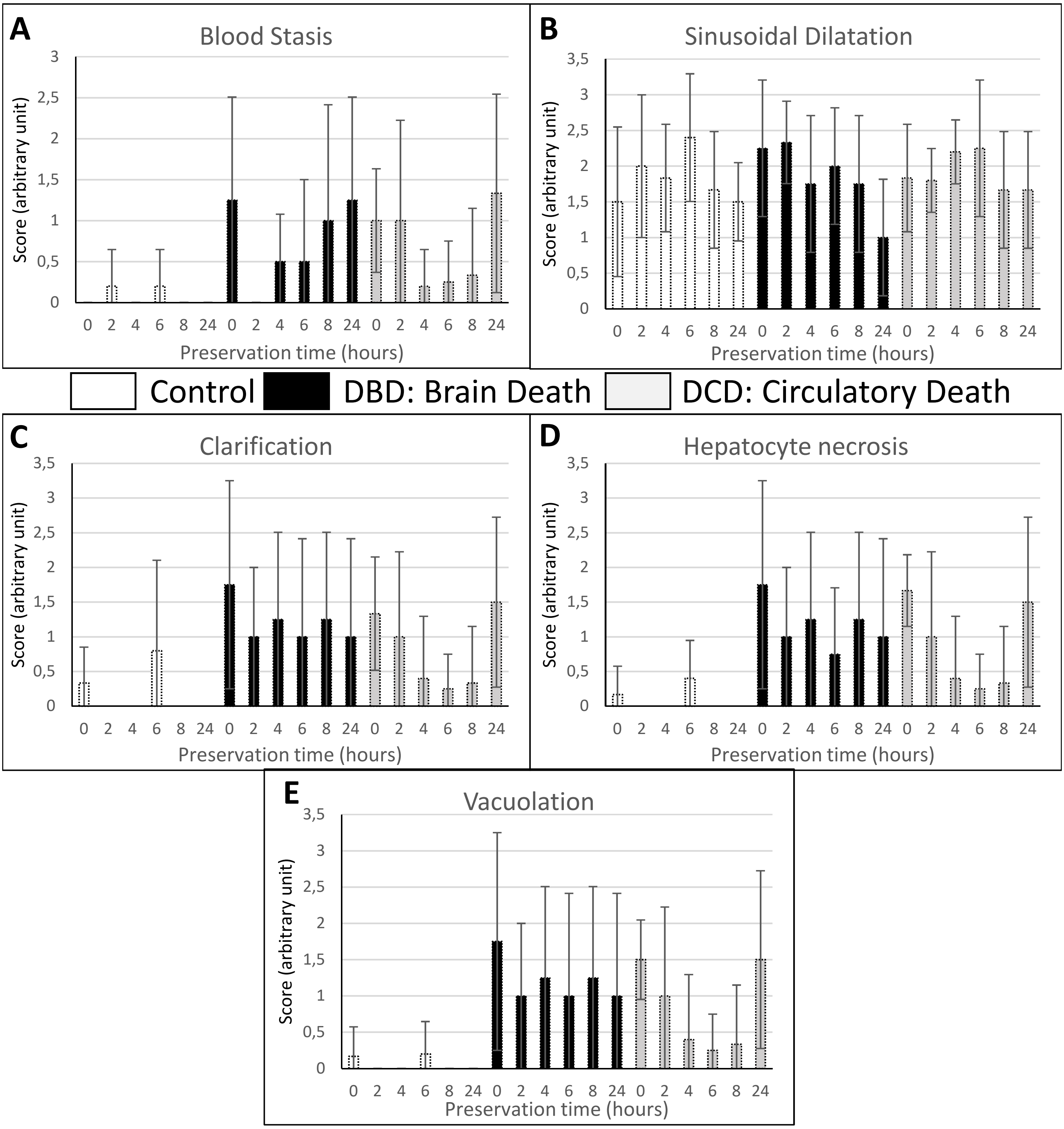

Supplementary Figure 2: Histological evaluation of preserved livers.
Formalin fixed liver samples collected at the indicated times were processes for HES and PAS staining and score according to the algorithm highlighted in Supplementary Figure 1. Five lesion types were analyzed: Blood Stasis, Vacuolation; Hepatocyte Necrosis, Clarification, and Sinusoid dilation. CTL group: gray bars; DBD group: black bars; DCD group: white bars. n=5.

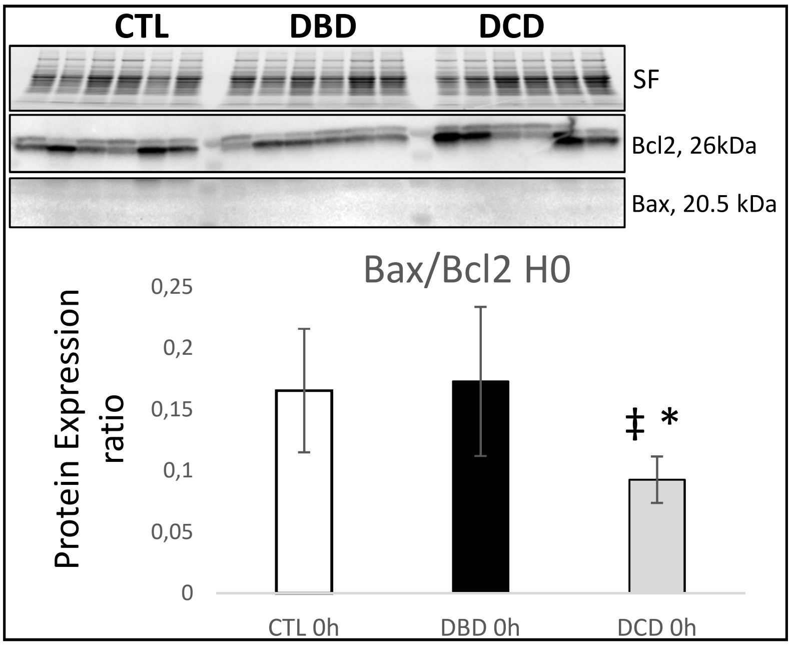

Supplementary Figure 3: Western blot evaluation of apoptosis.
Frozen samples were collected after organ rinsing and processes for western blot analysis. The signal is shown on top, with normalizing stain free blot (SF) shown underneath; quantification is shown at the bottom.
CTL group: gray bars; DBD group: black bars; DCD group: white bars. Statistics: shown are mean ± SEM, multiple comparison tests were performed using ANOVA Kruskal Wallis + Dunns Test, †: $p<0.05$ within group; *: $p<0.05$ to CTL at the same time; $p<0.05$ ‡: to DCD, at the same time. n=5.

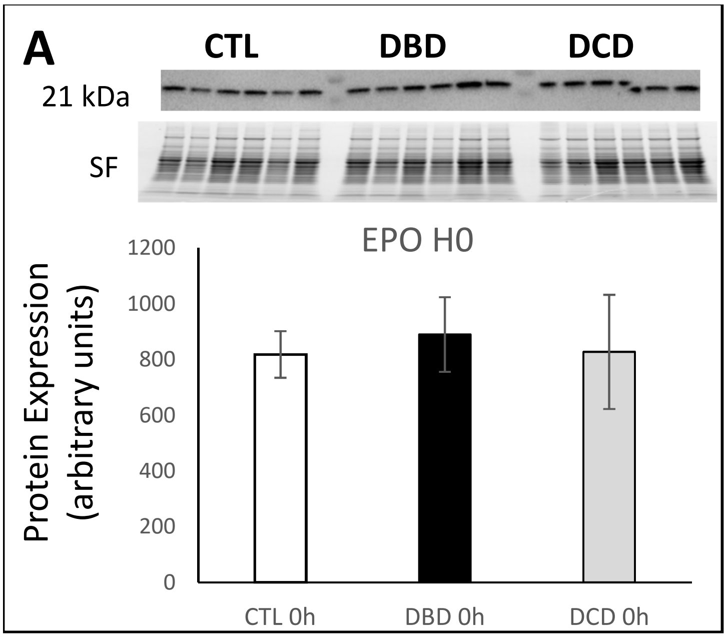

Supplementary Figure 4: Western Blot evaluation of EPO

Frozen samples were collected after organ rinsing and processes for western blot analysis. The signal is shown on top, with normalizing stain free blot (SF) shown underneath; quantification is shown at the bottom. CTL group: gray bars; DBD group: black bars; DCD group: white bars.

Key findings	Description
Hepatocyte necrosis	Signs of necrosis (peripheral nucleus, condensed chromatin, loss of membrane...) in hepatocyte cytoplasm
**Score**: None =0; <10% = 1; 10-20% = 2; >20% = 3	
Blood stasis	Spots with presence or infiltration by blood cells
**Score**: None =0; 1-2 = 1; 3-10 = 2; >10 = 3	
Vacuolation	Appearance of vacuoles in hepatocyte cytoplasm
**Score**: None =0; <10% = 1; 10-20% = 2; >20% = 3	
Ballooning degeneration	Loss of cytoplasm density
**Score**: None =0; <10% = 1; 10-20% = 2; >20% = 3	
Sinusoidal dilatation	
**Score**: None =0; <10% = 1; 10-20% = 2; >20% = 3	

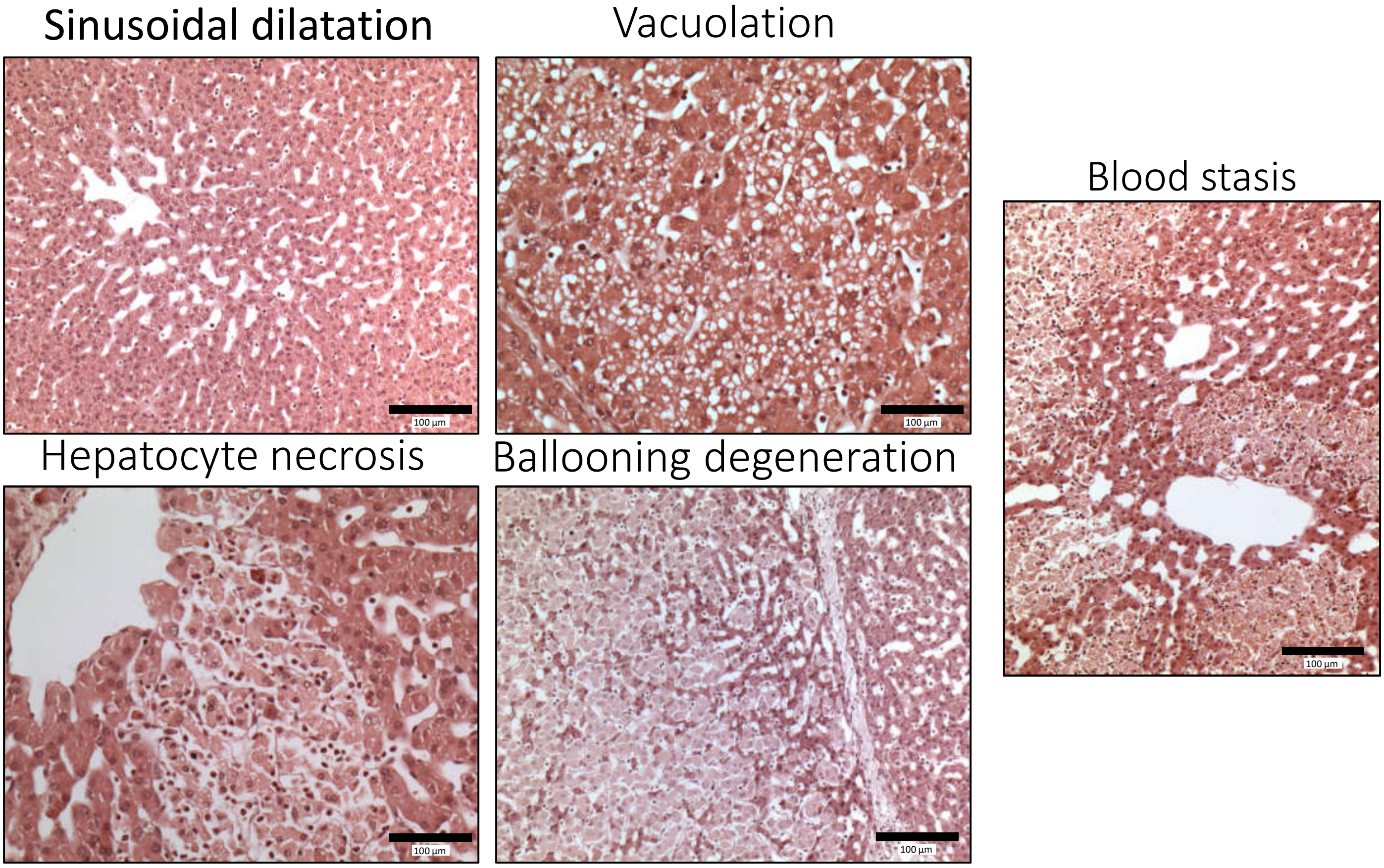

Supplementary Figure 5: Histological scoring

Top: Table detailing the scoring algorithm used for the histology, with each key finding, its definition, and the score. **Bottom**: representative images of each finding scored.
